# Meta-analysis and dose–response analysis of low-temperature stress effects on rice yield and physiological responses

**DOI:** 10.3389/fpls.2026.1775257

**Published:** 2026-06-09

**Authors:** Lixin Zhang, Jingya Zhou, Yanjie Lv, Jiani Li, Jiao Wang, Congling Zhu, Minjie Fu, Yongjun Wang

**Affiliations:** 1 College of Medicine, Yanbian University, Yanji, China; 2 College of Plant Science, Jilin University, Changchun, China; 3 College of Geography and Ocean Sciences, Yanbian University, Hunchun, China; 4 Institute of Agricultural Resources and Environment, Jilin Academy of Agricultural Sciences (Northeast China Innovation Center of Agricultural Science and Technology), Changchun, China; 5 College of Agronomy, Yanbian University, Yanji, China

**Keywords:** dose–response analysis, low temperature stress, meta-analysis, photosynthesis, physiological and biochemical responses, rice, yield

## Abstract

Low-temperature stress is a major environmental constraint on rice yield stability, considerable variation remains in the reported direction and magnitude of these effects. In this study, we conducted a systematic meta-analysis combined with dose–response analysis to evaluate the effects of low-temperature stress on rice yield formation, photosynthetic capacity, and physiological responses. The results showed that low-temperature stress significantly reduced yield-related traits in rice, with yield per plant and pollen viability decreasing by 10.58% and 21.75%, respectively. Photosynthetic performance was also markedly affected, as net photosynthetic rate (Pn), transpiration rate (Tr), and stomatal conductance (Gs) decreased by 26.67%, 10.93%, and 31.00%, respectively, while intercellular CO_2_ concentration (Ci) increased by 5.75%. Low-temperature stress also significantly induced the accumulation of osmotic regulatory substances and aggravated oxidative damage. Subgroup analysis and meta-regression indicated that the effects of low-temperature stress were jointly regulated by growth stage, stress temperature, and stress duration. Specifically, yield responses were more sensitive at the booting stage and heading–flowering stage, while Pn showed more pronounced responses to severe low temperature and prolonged stress. Dose–response analysis showed that most traits exhibited response turning points around 24 °C. Overall, this study quantitatively revealed the associations among yield reduction, photosynthetic inhibition, and physiological homeostasis imbalance in rice under low-temperature stress, providing comprehensive evidence for understanding the response patterns of rice to low temperature.

## 1 Introduction

Given the escalating climate variability and the growing frequency of extreme cold events, it is crucial to precisely assess the effects of low-temperature stress on rice production to safeguard global food security (Li et al., 2022). In comparison to other abiotic stresses, low temperature has the potential to quickly disrupt physiological and metabolic processes, leading to cold injury and presenting significant challenges to cropping systems and varietal adaptation (Kollist et al., 2019; Wu et al., 2022). Rice, a primary staple for over half of the global population (Zeng et al., 2017), exhibits high sensitivity to temperature fluctuations throughout its growth cycle, particularly during key reproductive stages such as panicle differentiation, heading–flowering, and grain filling, which are pivotal for yield formation (Arshad et al., 2017).

Exposure to low temperatures during reproductive development disrupts floral organ formation and panicle structure, resulting in reduced pollen viability, impaired fertilization, and spikelet sterility. These effects ultimately reduce grain number and thousand-grain weight, leading to significant yield losses and increased yield instability (Sun et al., 2025; Yang et al., 2024). With the increasing frequency of abnormal cold events, cold stress has become a major risk factor limiting the stability of rice production (Kang et al., 2022), underscoring the urgent need for systematic and quantitative assessments of its impact on yield formation.

At the physiological level, low temperature disrupts plant homeostasis via multiple interconnected pathways (Qian et al., 2024). Osmotic adjustment serves as a crucial protective strategy in rice, wherein the accumulation of proline, soluble sugars, and soluble proteins helps maintain cellular water potential and membrane stability, thereby enhancing cold tolerance (Guo et al., 2022a). Meanwhile, low temperature disrupts the balance between photosynthesis and respiration, leading to the excessive production of reactive oxygen species (ROS) and inducing lipid peroxidation and membrane damage (Han et al., 2020; Xu et al., 2024). Plants alleviate oxidative injury through the coordinated action of antioxidant enzyme systems and osmotic regulation, which together contribute to the preservation of metabolic function under cold stress (Hussain et al., 2018; Huy and Schlappi, 2021).

Low temperature also impairs photosynthetic efficiency, dry matter accumulation, and the activity of key metabolic enzymes, thereby limiting panicle development and grain filling capacity (Ma et al., 2025; Shi et al., 2022). Variations in the magnitude of temperature reduction and stress duration can induce varying degrees of osmotic regulation, oxidative damage, and antioxidant responses, leading to marked nonlinear changes in yield and physiological traits (Guo et al., 2025; Veresoglou and Begum, 2024). Furthermore, varietal differences, management practices, soil conditions, and microclimatic factors further modulate rice responses to low temperature, contributing to significant heterogeneity among studies (Pincebourde et al., 2016; Wang et al., 2025b).

Although numerous experimental studies have investigated the effects of low-temperature stress on rice growth and yield (Ai et al., 2023; Guo et al., 2022a), due to differences in temperature settings, classification of growth stages, stress duration, and measured indicators, the results of existing studies are difficult to directly compare and synthesize. In addition, most previous studies have adopted a “control–treatment” analytical framework, while relatively few have treated temperature as a continuous variable for dose–response analysis (Ali et al., 2021; Guo et al., 2024, 2025), which limits the ability to elucidate the nonlinear response of rice to low-temperature stress. To fill this gap and systematically address the variability among different studies, we used 24 °C as the reference temperature for data standardization, thereby reducing differences caused by inconsistent temperature settings across studies and improving the comparability of the results. Meanwhile, by combining meta-analysis with dose–response modeling, this study revealed the nonlinear effects of low temperature, treated as a continuous variable, on rice physiological traits, providing a quantifiable reference for assessing the risk of low-temperature stress.

## 2 Materials and methods

### 2.1 Data collection

A systematic literature search was performed in November 2025, utilizing the Web of Science and PubMed databases. The search query was: (“Rice” OR “Oryza sativa”) AND (“low temperature” OR “cold stress” OR “chilling” OR “cold tolerance”). The search period was limited from 1 January 2001 to 7 November 2025. No language or document type restrictions were applied during the search. Duplicates were removed using reference management software, and the remaining results were used for screening (**Supplementary Figure 1**).

To ensure the appropriateness of the data for meta-analysis, strict inclusion criteria were applied:(1) the experiment must include clearly defined low-temperature treatments and corresponding control groups with comparable outcomes; (2) studies must report the mean values, sample sizes, and measures of variability (standard deviation, SD, or standard error, SE), with at least three replicates per treatment; (3) in cases of duplicated or overlapping datasets, only the study providing the most comprehensive information was retained; (4) when a single publication contained multiple independent experiments, each experiment was treated as an independent study.

Based on these criteria, a total of 1,453 observations were extracted, covering low-temperature treatments applied during the tillering stage, booting stage, heading–flowering stage, and grain-filling stage of rice. These observations comprehensively documented the effects of low-temperature stress on rice yield, photosynthetic characteristics, and physiological and biochemical responses, providing a robust database for subsequent analyses.

All observations were classified into three categories comprising 18 response variables: (1) yield and quality-related traits: grain yield per plant (GY), seed setting rate (SSR), thousand-grain weight (TGW), pollen viability (PV), spikelet number per panicle (SNP); (2) photosynthesis-related traits: photosynthetic rate (Pn), intercellular CO_2_ concentration (Ci), transpiration rate (Tr), and stomatal conductance (Gs); (3) physiological response traits: proline (Pro), soluble sugar (SS), soluble protein (SP), electrolyte leakage (EL), malondialdehyde (MDA), superoxide anion (O_2_^-^), hydrogen peroxide (H_2_O_2_), superoxide dismutase (SOD), and peroxidase (POD).

When numerical data were presented graphically, values were extracted using GetData Graph Digitizer (version 2.26) to ensure data accuracy.

### 2.2 Meta-analysis

The analytical unit was defined as a single effect size jointly determined by the study, trait, stress temperature, stress duration, growth stage, and treatment–control combination. Considering that multiple non-independent effect sizes may be derived from the same study, we further applied a three-level random-effects meta-analysis model to distinguish within-study effect-size variation from between-study variation, thereby improving the robustness of heterogeneity estimation and effect-size inference. The meta-analysis followed a two-step procedure. First, the effect size and its variance were calculated for each independent study to standardize measurements across studies. Second, the overall effect size was estimated using a random-effects model. Effect sizes were quantified using the natural logarithm of the response ratio (lnR) (Zhou et al., 2022):


$$\ln R=\ln\left({\bar{X}}_{T}/{\bar{X}}_{C}\right)$$

Where ${\bar{X}}_{T}$ and ${\bar{X}}_{C}$ represent the mean values of the low-temperature treatment and control groups, respectively. The variance of lnR was calculated as:


$$\text{Var}\left(\ln R\right)=\frac{SD_{T}^{2}}{n_{T}{\bar{X}}_{T}^{2}}+\frac{SD_{C}^{2}}{n_{C}{\bar{X}}_{C}^{2}}$$

Where SD_T_ and n_T_ denote the standard deviation and sample size of the treatment group, and SD_C_ and n_C_ denote those of the control group. When variability was reported as standard error (SE), it was converted to SD using the following equation (Lv et al., 2025):


$$SD=SE\times \sqrt{n}$$

To facilitate interpretation, lnR values were further converted into percentage changes (Yuan et al., 2023), keeping two decimal places. The formula is as follows:


$$E=\left(e^{lnR}-1\right)\times 100\%$$

Given the variability in experimental materials, treatment regimes, and environmental conditions among studies, a random-effects model was employed to synthesize effect sizes (Barili et al., 2018). Because some traits were represented by a limited number of studies, further subgroup stratification could lead to unstable estimates and reduced statistical power (Gronsbell et al., 2025; Roever et al., 2015). Therefore, overall effect sizes were primarily analyzed for all traits. In addition, subgroup analyses and meta-regression were conducted for grain yield per plant (GY) and net photosynthetic rate (Pn), which had relatively concentrated datasets and clear data structures. Subgroups were defined based on stress period, stress duration, and stress temperature, with developmental-stage comparisons focusing on the booting stage, heading–flowering stage, and grain-filling stage. The residual heterogeneity among studies was assessed using the *Q_E_* test (Geng et al., 2017):


$$Q_{E}=\sum_{i=1}^{k}w_{i}{\left(y_{i}-{\hat{y}}_{i}\right)}^{2}$$

where k is the number of studies, w_i_ is the weight of the i-th effect size, *y_i_* is the effect size of the *i*-th study, and ${\hat{y}}_{i}$ e presents the effect size predicted by the model based on the moderator variables.

To evaluate the degree of variation in effect sizes among different studies, this study used *I^2^* 和 τ^2^ to quantify between-study heterogeneity (**Supplementary Table 1**). The normality of effect-size distributions was evaluated using the Kolmogorov–Smirnov, Shapiro–Wilk, D’Agostino–Pearson, and Anderson–Darling tests, complemented by Gaussian-fitted histograms for visual inspection (**Supplementary Figure 2**) (Gosselin, 2024; Le Boedec, 2016; Mishra et al., 2019). As effect sizes exhibited skewed distributions, weighted bootstrap resampling was applied to estimate 95% confidence intervals (CI) (Efron and Narasimhan, 2020). A low-temperature effect was considered statistically significant when the 95% CI did not include zero (Mathur and VanderWeele, 2020). Potential publication bias was assessed using Rosenthal’s fail-safe number (**Supplementary Table 2**). When the fail-safe number exceeded 5n + 10 (where n is the number of included studies), the effect size was considered robust against unpublished null results, indicating no significant publication bias (Kreplin et al., 2018; Rosenberg, 2005).The significance of the overall effect was assessed using the Chi-Square probability (Prob(Chi-Square)), whereas the significance within subgroups was evaluated using the random-effects probability (Prob(Rand)).

### 2.3 Dose-response curves

To characterize continuous responses of rice traits to temperature gradients under low-temperature stress, dose–response curves were constructed. Unlike conventional meta-analyses that classify temperature treatments into discrete categories, continuous temperature modeling allows a more accurate depiction of trait response trajectories (Shim and Lee, 2019; Xiong et al., 2017). Given that 24 °C appears most frequently in the database and lies at the lower boundary of the optimal growth temperature range for rice, it is commonly regarded as the critical threshold for low-temperature stress (Beesinger et al., 2022; El-Refaee et al., 2024; Hu et al., 2024; Ma et al., 2025). Therefore, 24 °C was selected as the reference temperature in this study, which not only aligns with the cold stress definition in the literature but also facilitates comparison with existing research results. To eliminate differences in measurement scales among studies and improve comparability, trait values were normalized (Poorter et al., 2019) using the following equation:


$$V_{i}^{Scaled}=\frac{V_{i}}{V_{ref}}$$

where V_i_ represents the observed value at temperature *i*, and *V_ref_* is the corresponding value at the reference temperature (24 °C). When studies did not directly report values at 24 °C, *V_ref_* was estimated by linear interpolation between adjacent temperature points to maintain data continuity and consistency (Larson et al., 2023). To examine the influence of reference temperature selection and interpolation processing on the normalized results, we further conducted a sensitivity analysis. After recalculating the normalized values using 20 °C as an alternative reference temperature, we found that the visualization trends under the two reference temperatures were generally consistent (**Supplementary Figure 3**), and the main turning points of trait responses both appeared around 24 °C. The paired-sample t-test showed no significant difference between the results obtained from the two normalization procedures (t = −1.05, *p* = 0.294), indicating that the choice of reference temperature did not significantly affect the overall results. Therefore, using 24 °C as the reference temperature is reasonable and robust. Statistical tests indicated that several traits deviated significantly from normality (**Supplementary Figure 4**), and no explicit theoretical models describing trait–temperature relationships were available. Therefore, a nonparametric local polynomial regression approach (LOESS) was applied to fit dose–response curves (Clarke and Richardson, 2021; Prata et al., 2021). Due to insufficient data coverage or limited temperature ranges, electrolyte leakage (EL), peroxidase activity (POD) could not form robust dose–response relationships. Consequently, the remaining 16 traits were retained for dose–response analysis to ensure reliability and comparability. All dose–response analyses were conducted using R version 4.5.2.

### 2.4 Statistical analysis

Effect sizes (lnR) were calculated using MetaWin version 2.1. Normality tests and distribution visualizations were performed in GraphPad Prism version 10. All statistical analyses and graphical outputs were generated using GraphPad Prism 10 and R version 4.5.2.

## 3 Results

### 3.1 Effects of low temperature on rice yield

**Figure 1:**
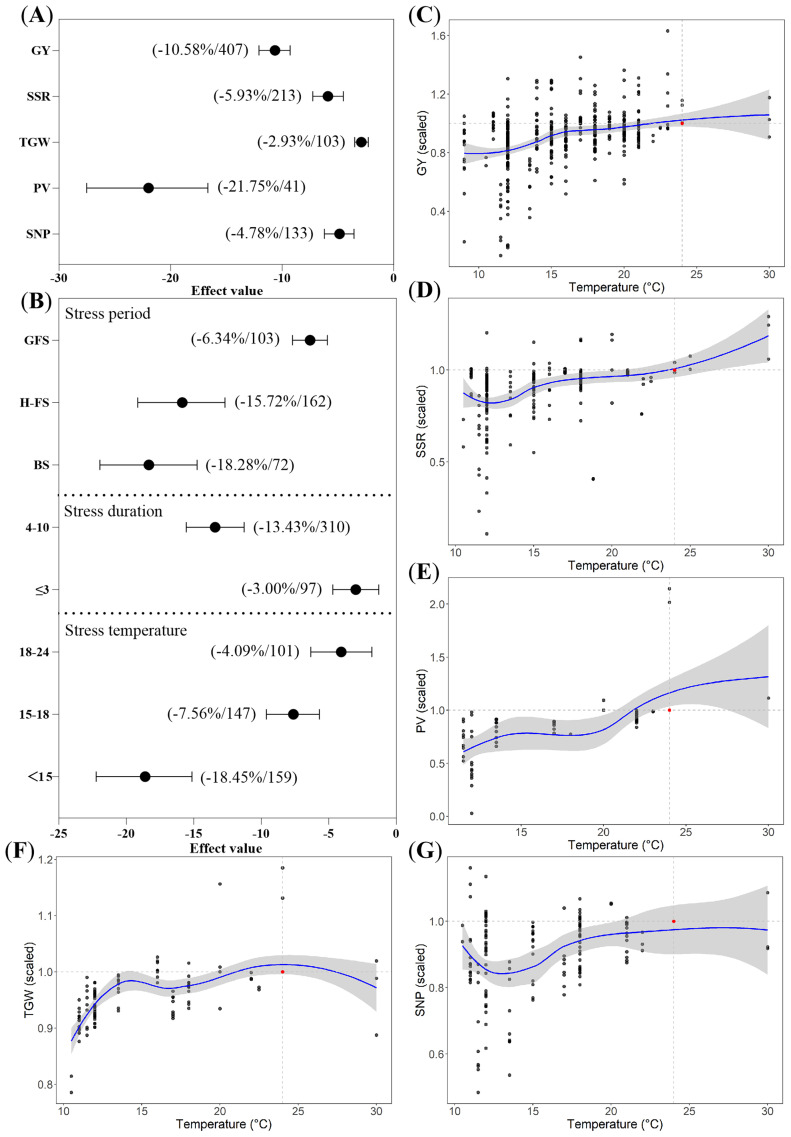
Effects of low temperature on rice yield and yield-related traits. **(A)** Overall effects of low-temperature stress on yield-related traits in rice based on meta-analysis; **(B)** Effects of low temperature on grain yield per plant under different stress conditions, including growth stage, stress duration, and temperature intensity; **(C–G)** Dose–response relationships between decreasing temperature and grain yield per plant (GY), seed-setting rate (SSR), pollen viability (PV), thousand-grain weight (TGW), and spikelet number per panicle (SNP). Stress period: BS, booting stage; H-FS, heading–flowering stage; GFS, grain-filling stage. Stress duration: ≤3 d and 4–10 d indicate different stress duration categories. Stress temperature: 18–24 °C, 15–24 °C, and<15 °C indicate different stress temperature ranges. In the dose–response curves, the dashed line indicates the reference temperature of 24 °C.

Overall, the meta-analysis showed that low-temperature stress significantly inhibited rice yield formation (**Supplementary Tables 2** and **3**). Compared with the control, grain yield per plant, seed-setting rate, and thousand-grain weight decreased by 10.58% (95% CI: −12.07% to −9.28%), 5.93% (95% CI: −7.25% to −4.51%), and 2.93% (95% CI: −3.50% to −2.28%), respectively. Meanwhile, pollen viability and spikelet number per panicle were also significantly reduced. Among these traits, pollen viability showed the greatest decline, decreasing by 21.75% (95% CI: −27.52% to −16.66%), while spikelet number per panicle decreased by 4.78% (95% CI: −6.21% to −3.56%) (**Figure 1A**).

Subgroup analysis revealed pronounced stage-dependent and intensity-dependent effects of low-temperature stress on grain yield per plant (**Figure 1B**). The booting stage and heading–flowering stage were the most sensitive stages for yield response to low-temperature stress, with grain yield per plant decreasing by 18.28% (95% CI: −21.96% to −14.75%) and 15.72% (95% CI: −19.15% to −12.69%), respectively. These reductions were greater than that observed at the grain-filling stage, where grain yield per plant decreased by 6.34% (95% CI: −7.69% to −5.11%). In addition, yield losses became more severe as stress duration increased and temperature decreased. When the temperature was below 15 °C, grain yield per plant decreased by 18.45% (95% CI: −22.23% to −15.13%). These results indicate that the negative effects of low temperature on rice yield are determined not only by the occurrence of stress, but also by the growth stage, stress duration, and temperature intensity.

Dose–response analysis showed that the effects of low-temperature stress on yield-related traits were clearly nonlinear (**Figures 1C–G**). Grain yield per plant (GY), seed-setting rate (SSR), pollen viability (PV), thousand-grain weight (TGW), and spikelet number per panicle (SNP) exhibited relatively consistent response turning points around 24 °C. When the temperature was below this level, all yield-related indicators decreased markedly with decreasing temperature, whereas at 24 °C and above, these indicators increased to varying degrees. This result suggests that the temperature range around 24 °C may represent a sensitive transition zone for the response of rice yield formation to low temperature, indicating that the effects of low-temperature stress may have threshold-like characteristics.

**Figure 2:**
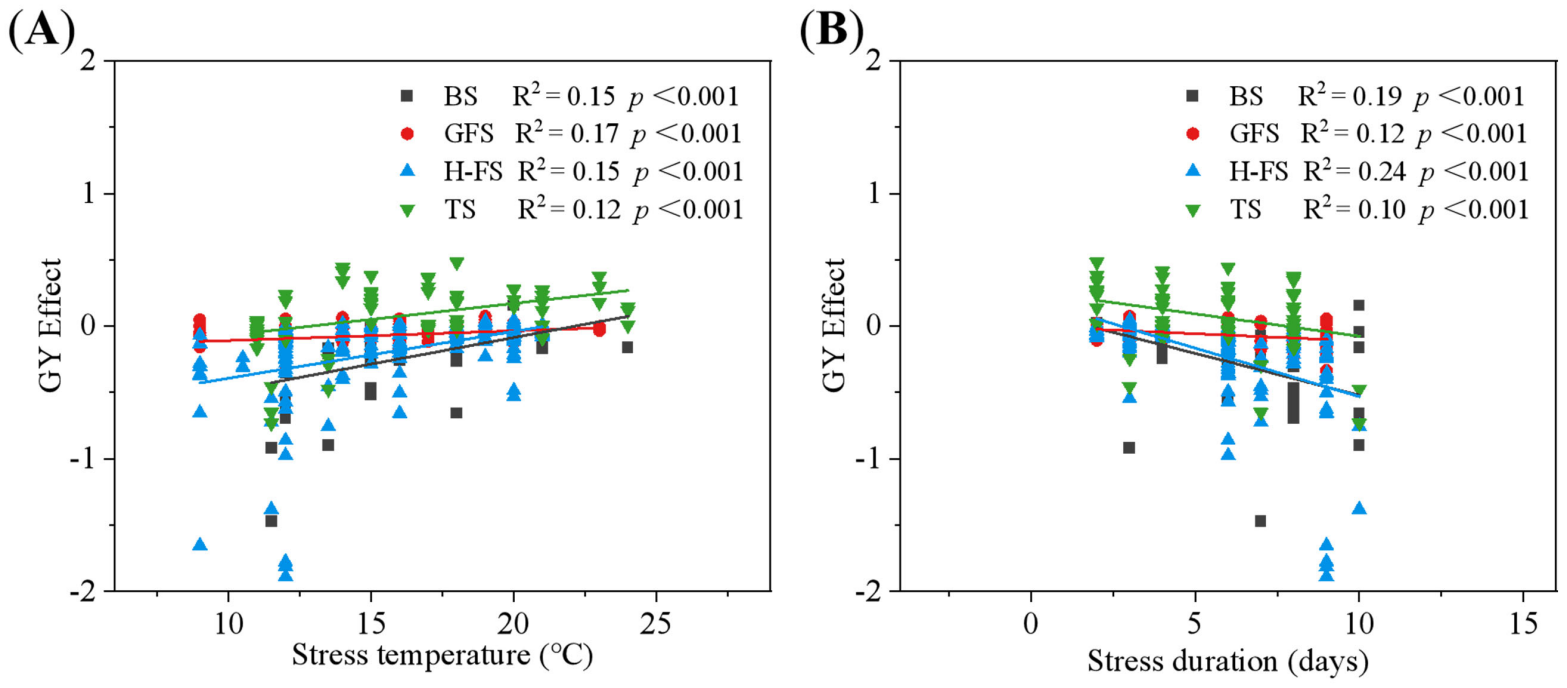
Meta-regression analysis of the effects of stress temperature and stress duration on grain yield per plant at different growth stages. **(A)** Relationship between stress temperature and the effect size of grain yield per plant; **(B)** Relationship between stress duration and the effect size of grain yield per plant. Stress period: TS, tillering stage; BS, booting stage; H-FS, heading–flowering stage; GFS, grain-filling stage.

Meta-regression analysis further showed that the effects of low-temperature stress on grain yield per plant varied markedly across growth stages with changes in stress temperature and stress duration (**Figures 2A, B**). Along the stress temperature gradient, the regression slopes across growth stages followed the order BS > H-FS > TS > GFS, indicating that the yield effect at the booting stage was most sensitive to temperature changes, followed by the heading–flowering stage and tillering stage, whereas the response at the grain-filling stage was relatively weak. In other words, as stress temperature decreased, the increase in yield loss was greatest at the booting stage, suggesting that this stage represents a key sensitive period during which low temperature affects yield formation. Along the stress duration gradient, the slopes across growth stages followed the order H-FS > BS > TS > GFS, indicating that prolonged low-temperature stress exerted the strongest inhibitory effect on yield at the heading–flowering stage, followed by the booting stage and tillering stage. These results suggest that the effect of low-temperature stress on yield is determined not only by temperature intensity, but also by stress duration and the timing of stress occurrence. Specifically, the booting stage was more susceptible to low-temperature intensity, whereas the heading–flowering stage was more sensitive to stress duration.

### 3.2 Effects of low temperature on photosynthetic performance in rice

**Figure 3:**
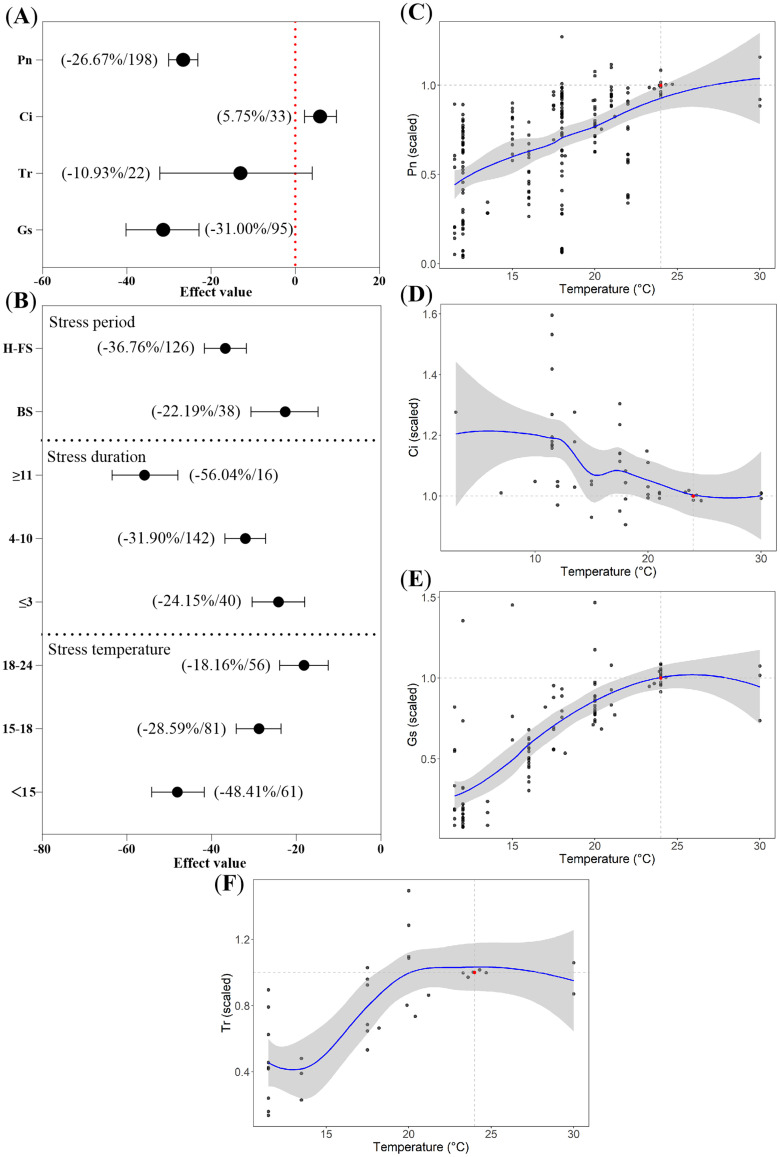
Effects of low temperature on photosynthetic characteristics in rice. **(A)** Overall effects of low-temperature stress on photosynthesis-related parameters in rice based on meta-analysis; **(B)** Effects of low temperature on rice photosynthetic performance under different stress conditions, including growth stage, stress duration, and temperature intensity; **(C–F)** Dose–response relationships between decreasing temperature and net photosynthetic rate (Pn), intercellular CO_2_ concentration (Ci), transpiration rate (Tr), and stomatal conductance (Gs). Stress period: BS, booting stage; H-FS, heading–flowering stage. Stress duration: ≤3 d, 4–10 d, and ≥11 d indicate different stress duration categories. Stress temperature: 18–24 °C, 15–24 °C, and<15 °C indicate different stress temperature ranges. In the dose–response curves, the dashed line indicates the reference temperature of 24 °C.

Overall analysis showed that low-temperature stress significantly inhibited photosynthetic processes in rice (**Figure 3A**). Compared with the control, net photosynthetic rate (Pn), transpiration rate (Tr), and stomatal conductance (Gs) decreased by 26.67% (95% CI: −30.06% to −23.12%), 10.93% (95% CI: −32.15% to −4.03%), and 31.00% (95% CI: −40.18% to −22.87%), respectively. In contrast, intercellular CO_2_ concentration (Ci) increased by 5.75% (95% CI: 2.21% to 9.80%), indicating that low-temperature stress not only weakened leaf CO_2_ exchange capacity but may also have constrained photosynthetic carbon assimilation.

Subgroup analysis of Pn further showed that the inhibitory effects of low temperature on rice photosynthetic capacity were strongly dependent on growth stage, stress duration, and temperature intensity (**Figure 3B**). Among different growth stages, Pn decreased by 22.19% (95% CI: −30.70% to −14.86%) and 36.76% (95% CI: −41.70% to −31.78%) at the booting stage and heading–flowering stage, respectively, indicating that photosynthesis during the reproductive growth period was more sensitive to low-temperature stress. As stress duration increased, the reduction in Pn became progressively greater, increasing from 24.15% (95% CI: −30.41% to −18.00%) under ≤3 d of stress to 31.90% (95% CI: −36.85% to −27.25%) under 4–10 d of stress, and reaching 56.04% (95% CI: −63.51% to −47.95%) when stress lasted for ≥11 d. In addition, Pn showed a clear gradient response to stress temperature. Under 18–24 °C, 15–18 °C, and<15 °C conditions, Pn decreased by 18.16% (95% CI: −23.91% to −12.44%), 28.59% (95% CI: −34.18% to −23.57%), and 48.41% (95% CI: −54.15% to −41.73%), respectively, indicating that lower temperatures caused stronger inhibition of photosynthetic rate.

Dose–response analysis further supported these findings (**Figures 3C–F**). As temperature decreased, Pn, Tr, and Gs all showed nonlinear decreasing trends, whereas Ci exhibited the opposite increasing trend. All photosynthesis-related indicators showed response changes around 24 °C, and the inhibitory effects on photosynthesis became more pronounced below this temperature, suggesting that 24 °C may serve as an important reference temperature for evaluating the response of rice photosynthetic processes to low-temperature stress.

**Figure 4:**
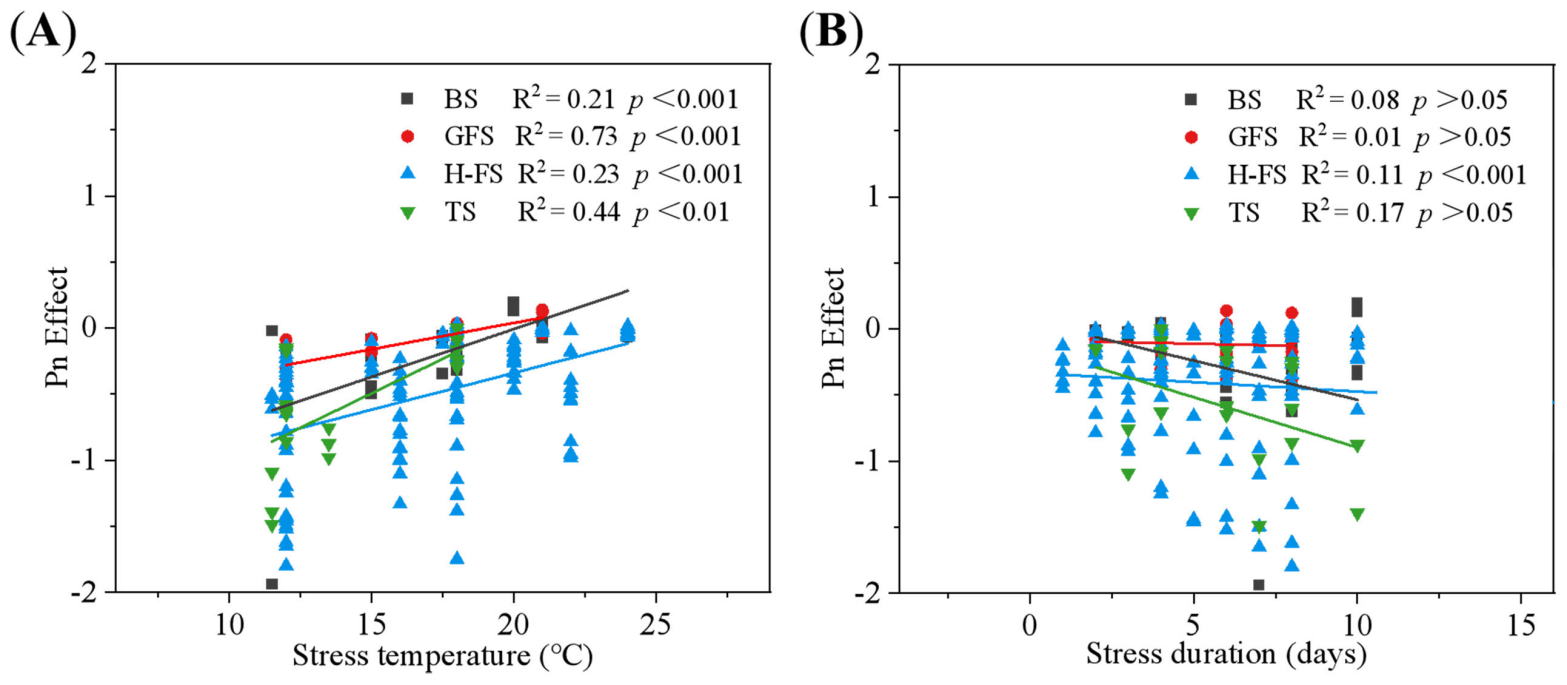
Meta-regression analysis of the effects of stress temperature and stress duration on net photosynthetic rate at different growth stages. **(A)** Relationship between stress temperature and the effect size of net photosynthetic rate; **(B)** Relationship between stress duration and the effect size of net photosynthetic rate. Stress period: TS, tillering stage; BS, booting stage; H-FS, heading–flowering stage; GFS, grain-filling stage.

Meta-regression analysis showed that the effect of low-temperature stress on net photosynthetic rate (Pn) in rice was jointly regulated by stress temperature, stress duration, and growth stage (**Figures 4A, B**). Along the stress temperature gradient, the response slopes of Pn effect sizes across growth stages followed the order BS > TS > H-FS > GFS, indicating that Pn at the booting stage was most sensitive to decreasing temperature, followed by the tillering stage, whereas the response at the grain-filling stage was the weakest (**Figure 4A**). Along the stress duration gradient, the response slopes followed the order TS > BS > H-FS > GFS, suggesting that prolonged stress duration exerted the strongest inhibitory effect on Pn at the tillering stage, followed by the booting stage (**Figure 4B**). Overall, the effect of low-temperature stress on Pn showed clear stage dependence: the booting stage was mainly characterized by high sensitivity to low-temperature intensity, whereas the tillering stage was more sensitive to stress duration.

### 3.3 Effects of low temperature on physiological responses in rice

**Figure 5:**
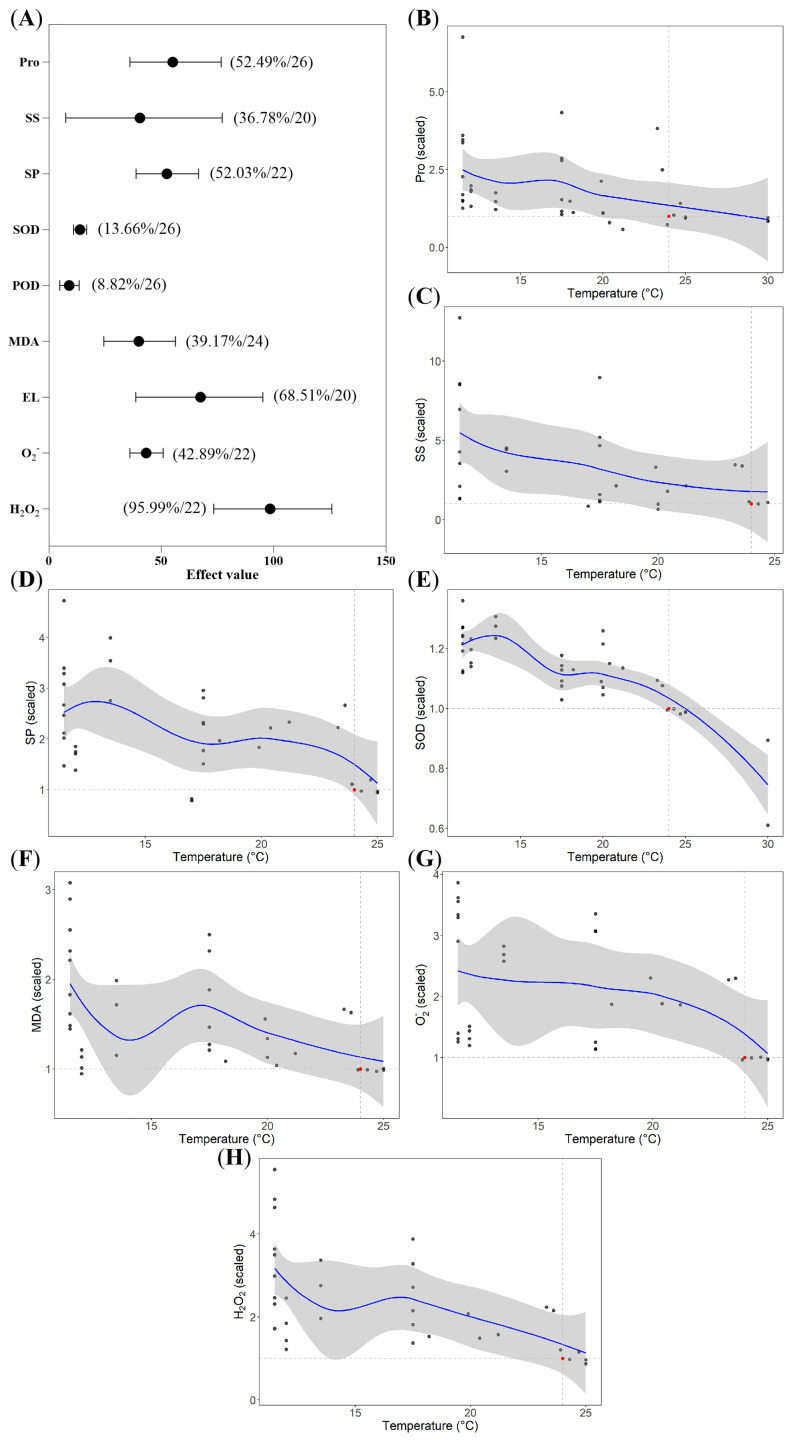
Effects of low temperature on physiological responses in rice. **(A)** Overall effects of low-temperature stress on physiological responses in rice; **(B–H)** Dose–response relationships between decreasing temperature and proline (Pro), soluble sugar (SS), soluble protein (SP), superoxide dismutase (SOD), malondialdehyde (MDA), superoxide anion (O_2_^-^), and hydrogen peroxide (H_2_O_2_). In the dose–response curves, the dashed line indicates the reference temperature of 24 °C.

.Low-temperature stress significantly induced osmotic adjustment and antioxidant defense responses in rice (**Figure 5A**). Compared with the control, proline, soluble sugar, and soluble protein contents increased by 52.49% (95% CI: 36.00% to 76.65%), 36.78% (95% CI: 7.39% to 77.07%), and 52.03% (95% CI: 38.75% to 66.63%), respectively. Meanwhile, the activities of superoxide dismutase (SOD) and peroxidase (POD) were also significantly enhanced, increasing by 13.66% (95% CI: 10.96% to 16.82%) and 8.82% (95% CI: 4.77% to 13.45%), respectively. These results indicate that low-temperature stress can activate the accumulation of osmoprotective substances and the antioxidant enzyme defense system in rice.

Low-temperature stress also significantly aggravated cellular oxidative damage (**Figure 5A**). Compared with the control, malondialdehyde (MDA), electrolyte leakage, superoxide anion (O_2_^-^), and hydrogen peroxide (H_2_O_2_) increased by 39.17% (95% CI: 24.42% to 56.25%), 68.51% (95% CI: 38.65% to 95.21%), 42.89% (95% CI: 36.03% to 50.83%), and 95.99% (95% CI: 73.38% to 125.94%), respectively. Among these indicators, the increases in H_2_O_2_ and electrolyte leakage were particularly pronounced, suggesting that low temperature not only promoted the accumulation of reactive oxygen species but also intensified membrane lipid peroxidation and cellular membrane damage.

Dose–response analysis further showed that osmotic adjustment substances, antioxidant enzyme activities, and oxidative damage indicators all tended to increase as temperature decreased (**Figures 5B–H**). This result indicates that low-temperature-induced defense responses and damage accumulation were enhanced simultaneously, reflecting the dual process in which rice undergoes both adaptive protective responses and aggravated cellular damage under low-temperature stress.

## 4 Discussion

### 4.1 Low temperature reduces rice yield

This study showed that low-temperature stress significantly reduced rice yield and yield-related traits, including grain yield per plant (GY), seed-setting rate (SSR), thousand-grain weight (TGW), pollen viability (PV), and spikelet number per panicle (SNP) (**Figure 1A**). These results indicate that the inhibitory effect of low-temperature stress on rice yield formation is not driven by a single yield component, but involves multiple processes, including reproductive development, seed setting, and grain formation. Among these traits, the marked decline in pollen viability may represent an important upstream cause of yield loss. Pollen development, pollination, and fertilization are highly sensitive to low temperature. Low temperature may disrupt anther development, pollen maturation, pollen germination, and pollen tube elongation, thereby reducing fertilization success and ultimately leading to a decline in seed-setting rate. Meanwhile, the reduction in thousand-grain weight indicates that low temperature affects not only the seed-setting process but also further limits grain filling and dry matter accumulation (Unan et al., 2022; Yang et al., 2024; Zhang et al., 2021). Therefore, the negative effect of low-temperature stress on rice yield is systemic, mainly characterized by continuous inhibition from pollen viability and seed-setting rate to grain weight (Wang et al., 2021, 2020).

Subgroup analysis further showed that sensitivity to low temperature differed significantly among growth stages (**Figure 1B**). Low-temperature stress at the booting stage and heading–flowering stage had the strongest inhibitory effects on grain yield per plant, with yield reductions of 18.28% and 15.72%, respectively, whereas the reduction at the grain-filling stage was relatively small, at only 6.34%. This result suggests that the sensitive window of rice yield response to low temperature is mainly concentrated during the reproductive growth stage. The booting stage is a critical period for panicle differentiation, anther development, and pollen formation, during which low temperature can easily induce pollen abortion, spikelet degeneration, and abnormal reproductive organ development. The heading–flowering stage is directly associated with pollen release, pollination, fertilization, and spikelet fertility, and low temperature can cause seed-setting failure by reducing pollen viability, delaying flowering, or obstructing fertilization (Ali et al., 2021; Arshad et al., 2017; El-Refaee et al., 2024; Vargas et al., 2021). Therefore, the booting stage and heading–flowering stage can be regarded as key sensitive windows during which low temperature affects rice yield formation. In contrast, although low temperature during the grain-filling stage can still affect photosynthesis, assimilate transport, and grain filling, its effect on final grain yield per plant is relatively weaker because pollen development and fertilization have largely been completed by this stage (Arshad et al., 2017; Ali et al., 2021).

In addition, the duration and intensity of low-temperature stress further amplified the risk of yield loss. Overall, longer stress duration and lower temperature were associated with greater yield reductions in rice (**Figure 1B**). Short-term low temperature may cause only temporary physiological inhibition, whereas medium- to long-term low temperature is more likely to continuously interfere with photosynthesis, carbon assimilation, pollen development, and assimilate transport, thereby leading to irreversible yield losses. In terms of temperature intensity, the largest yield reduction occurred under temperatures below 15 °C, indicating that severe low temperature has a stronger destructive effect on rice yield formation. By contrast, the effects under 15–18 °C and 18–24 °C were relatively weaker, suggesting that rice may have a certain buffering capacity against mild low-temperature stress (Ali et al., 2021; El-Refaee et al., 2024; Unan et al., 2022). The response turning point around 24 °C observed in the dose–response analysis also supports this interpretation, suggesting that 24 °C can serve as an important reference temperature for identifying sensitive responses to low temperature in this study. Taken together, the effect of low temperature on rice yield is jointly determined by growth stage, temperature intensity, and stress duration. Low-temperature exposure during the reproductive growth stage, especially severe or prolonged low temperature at the booting stage and heading–flowering stage, is the main cause of significant yield reduction.

### 4.2 Low temperature impairs photosynthetic performance in rice

Photosynthesis is the basis of crop dry matter accumulation and yield formation (Gong et al., 2023). In this study, low-temperature stress significantly decreased net photosynthetic rate (Pn), transpiration rate (Tr), and stomatal conductance (Gs), while increasing intercellular CO_2_ concentration (Ci) (**Figure 3A**). This response pattern indicates that low temperature not only weakened leaf gas exchange capacity but also markedly restricted photosynthetic carbon assimilation (Suganami et al., 2021). When Pn and Gs decreased simultaneously, the increase in Ci suggests that the limitation of photosynthesis under low temperature cannot be simply attributed to insufficient CO_2_ supply caused by stomatal closure. Instead, it is more likely associated with enhanced non-stomatal limitation, such as reduced mesophyll carboxylation capacity, impaired photosynthetic electron transport, or decreased activities of enzymes involved in carbon assimilation (Wada et al., 2019; Xu et al., 2023; Zhang et al., 2025). Therefore, the inhibition of the rice photosynthetic system by low temperature is comprehensive, involving not only decreases in stomatal conductance and transpiration but also impairment of internal carbon fixation capacity in leaves.

Subgroup analysis further showed that the inhibitory effect of low temperature on rice photosynthetic capacity was clearly growth stage-dependent and dose-dependent. Among different growth stages, the booting stage and heading–flowering stage were more sensitive to low temperature, with Pn decreasing by 22.19% and 36.76%, respectively. This indicates that the photosynthetic system during the reproductive growth period is more vulnerable to low-temperature disturbance. The booting stage and heading–flowering stage are critical periods for panicle differentiation, pollen development, flowering, fertilization, and early grain formation, during which the demand for photosynthate supply and assimilate transport is high. The reduction in Pn caused by low temperature during these stages may directly weaken carbon source supply, thereby affecting pollen viability, seed-setting rate, and grain formation (Li et al., 2024; Shi et al., 2023; Xu et al., 2023). In addition, Pn showed clear gradient responses to both stress duration and temperature intensity. As stress duration increased, the reduction in Pn increased from 24.15% under ≤3 d of stress to 31.90% under 4–10 d of stress, and reached 56.04% when stress lasted for ≥11 d. When the temperature was below 15 °C, Pn showed the greatest decline, reaching 48.41%, whereas it decreased by 28.59% and 18.16% under 15–18 °C and 18–24 °C, respectively. These results indicate that stronger low-temperature stress and longer exposure cause more severe inhibition of the photosynthetic system, potentially converting short-term reversible photosynthetic suppression into more pronounced physiological dysfunction.

Low temperature may restrict rice photosynthesis through multiple pathways. On the one hand, low temperature can reduce photosynthetic electron transport efficiency and decrease ATP and NADPH supply, thereby weakening the light reaction process (Shi et al., 2022; Wada et al., 2019; Yamori et al., 2016). On the other hand, low temperature may also inhibit the activities of key photosynthetic enzymes, such as Rubisco and ATP synthase, reducing carboxylation efficiency and carbon fixation capacity (Caine et al., 2019; Salesse-Smith et al., 2025). However, the increase in Ci suggests that non-stomatal limitation may be more critical in this study; that is, CO_2_ was not substantially insufficient due to stomatal closure but accumulated inside leaves because of reduced carbon fixation capacity (Xu et al., 2023). Since photosynthetic efficiency directly determines dry matter accumulation and carbon supply for grain filling, long-term or severe low-temperature inhibition of the photosynthetic system may further affect rice yield and quality formation (Shi et al., 2022; Wang et al., 2023b). In this study, the significant decreases in Pn, Tr, and Gs were consistent with the reductions in grain yield per plant, thousand-grain weight, and starch content, indicating that impaired photosynthetic carbon assimilation may be one of the important physiological bases by which low temperature reduces rice yield and quality (Salesse-Smith et al., 2025; Suganami et al., 2021; Wada et al., 2019).

### 4.3 Low temperature modulates osmotic adjustment, antioxidant enzyme activities, and oxidative stress responses in rice

Plant adaptation mechanisms to low-temperature stress generally include osmotic adjustment and antioxidant defense (de Freitas et al., 2019). In this study, low temperature significantly promoted the accumulation of osmotic adjustment substances in rice, including proline (Pro), soluble sugar (SS), and soluble protein (SP), while also enhancing the activities of superoxide dismutase (SOD) and peroxidase (POD) (**Figure 5A**). These changes indicate that rice can actively initiate protective physiological responses under low-temperature stress to maintain cellular osmotic balance and redox homeostasis. Osmotic adjustment substances can enhance cold tolerance by reducing cellular osmotic potential, stabilizing protein structures, protecting membrane systems, and alleviating low-temperature-induced cellular dehydration (Goswami et al., 2022; Raza et al., 2023). The antioxidant enzyme system reduces membrane lipid peroxidation and maintains cellular function by scavenging excessive reactive oxygen species (Shahzad et al., 2024; Wang et al., 2023b). Therefore, the increases in Pro, SS, SP, SOD, and POD can be regarded as adaptive defense responses of rice to low-temperature stress.

However, this study also observed significant increases in malondialdehyde (MDA), electrolyte leakage (EL), superoxide anion (O_2_^-^), and hydrogen peroxide (H_2_O_2_) (**Figure 5A**), indicating that the defense responses induced by low temperature did not fully offset cellular damage caused by oxidative stress (de Freitas et al., 2019; Guo et al., 2022b; Shi et al., 2024). MDA is an important product of membrane lipid peroxidation, while increased EL reflects damage to cellular membrane integrity. The accumulation of O_2_^-^ and H_2_O_2_ further indicates that ROS production under low temperature exceeded the scavenging capacity of the antioxidant system (Bala et al., 2025; Shahandashti et al., 2013; Shi et al., 2024). This suggests that the low-temperature response of rice has a dual nature: on the one hand, plants enhance tolerance through osmotic adjustment and antioxidant enzyme systems; on the other hand, when low-temperature stress is severe or prolonged, the defense system may become saturated, and excessive ROS accumulation can still disrupt membrane structures, protein function, and cellular metabolic homeostasis (Cao et al., 2025; Raza et al., 2022; Wang et al., 2023a).

Further integration with the photosynthesis and yield results of this study indicates that the effects of low temperature on rice are not caused by damage to a single physiological process, but rather by the coordinated action of multiple pathways. Low temperature significantly inhibited Pn, Tr, and Gs, reducing the carbon source supply required for grain filling. Meanwhile, low-temperature-induced accumulation of osmotic adjustment substances and enhancement of antioxidant defense may redirect more metabolic resources toward stress mitigation and cellular protection rather than starch synthesis and grain filling (Ma et al., 2025; Shi et al., 2022; Wang et al., 2023b; Wang et al., 2025a). In addition, as ROS accumulation and membrane damage intensify, photosynthetic tissues and cellular metabolic functions may be further impaired, which could further weaken carbon assimilation, assimilate transport, and grain development (Faisal et al., 2023; Guo et al., 2022a). Therefore, the declines in rice yield and quality caused by low temperature can be understood as the combined result of reduced photosynthetic carbon supply, metabolic resource redistribution, and cumulative oxidative damage. This mechanism is consistent with the decreases in grain yield per plant, seed-setting rate, and starch content, as well as the simultaneous increases in defense- and damage-related indicators observed in this study, indicating that the effects of low-temperature stress on rice yield and quality are systematic and multilayered (Shahzad et al., 2024; Wang et al., 2023a).

### 4.4 Limitations and future perspectives

Although this study integrated 1,453 data records from two commonly used databases and systematically quantified the effects of low-temperature stress on rice yield, photosynthesis, osmotic adjustment, and antioxidant defense-related indicators, several aspects of the current evidence base still warrant further improvement in future research. First, the data used in this study were mainly obtained from two databases, which cover the major literature sources related to the effects of low-temperature stress on rice yield and physiological responses. However, there remains room to include regional studies, dissertations, conference papers, and other forms of grey literature. Therefore, future studies could further expand database sources and document types, and incorporate more studies from different ecological regions, cultivation systems, and climatic backgrounds to improve the coverage of evidence and the generalizability of the conclusions. Second, existing studies mainly focus on phenotypic, physiological, and biochemical indicators, while evidence regarding the molecular regulatory relationships among photosynthetic carbon assimilation, antioxidant defense, grain filling, and starch accumulation under low-temperature stress remains insufficient. Future research could integrate transcriptomic, proteomic, metabolomic, and enzyme activity analyses to further clarify the key mechanisms by which low temperature affects rice yield and quality formation, thereby providing a more reliable basis for breeding cold-tolerant varieties and optimizing cultivation management.

## 5 Conclusions

Through meta-analysis and dose–response modeling, this study quantitatively evaluated the effects of low-temperature stress on rice yield formation, photosynthetic capacity, and physiological responses. The results showed that low temperature significantly reduced grain yield per plant, seed-setting rate, thousand-grain weight, pollen viability, and spikelet number per panicle, while simultaneously inhibiting photosynthesis and aggravating oxidative damage. Subgroup analysis and meta-regression further indicated that the effects of low temperature were jointly regulated by growth stage, stress temperature, and stress duration. Among these factors, the booting stage and heading–flowering stage were more sensitive in terms of yield formation, whereas net photosynthetic rate showed more pronounced responses to severe low temperature and prolonged stress. Dose–response analysis showed that most traits exhibited response turning points around 24 °C, suggesting that this temperature may serve as an important reference point for identifying low-temperature-sensitive responses in this study. Overall, this study provides comprehensive evidence for revealing the quantitative relationships among yield reduction, photosynthetic inhibition, and physiological homeostasis imbalance in rice under low-temperature stress.

## Data Availability

The raw data supporting the conclusions of this article will be made available by the authors, without undue reservation.

## Glossary

GY: grain yield per plant
SSR: seed setting rate
TGW: thousand-grain weight
PV: pollen viability
SNP: spikelet number per panicle
AP: amylopectin content
TS: total starch content
Pn: net photosynthetic rate
Ci: intercellular CO_2_ concentration
Tr: transpiration rate
Gs: stomatal conductance
Pro: proline
SS: soluble sugar
SP: soluble protein
EL: electrolyte leakage
MDA: malondialdehyde
O_2_^-^: superoxide anion
H_2_O_2_: hydrogen peroxide
SOD: superoxide dismutase
POD: peroxidase
