# Supplementary material for: Meta-analysis and dose–response analysis of low-temperature stress effects on rice yield and physiological responses
Source: Front Plant Sci. 2026 Jun 9;17:1775257. doi: 10.3389/fpls.2026.1775257 (PMC13288079; doi:10.3389/fpls.2026.1775257)
Supplement: Supplementary file 1 [file Table1.docx]

Support information

**Meta-analysis and dose–response analysis of low-temperature stress effects on rice yield and physiological responses**

Lixin Zhang, Jingya Zhou, Yanjie Lv, Jiani Li, Jiao Wang, Congling Zhu, Minjie Fu*, Yongjun Wang*

Minjie [Fu](mailto:Fu(fuminjie@163.com);)

Email: fuminjie@163.com

Yongjun Wang

Email: yjwang2004@126.com

**This file includes:**

Figure S1 to S4;

Tables S1 to S3


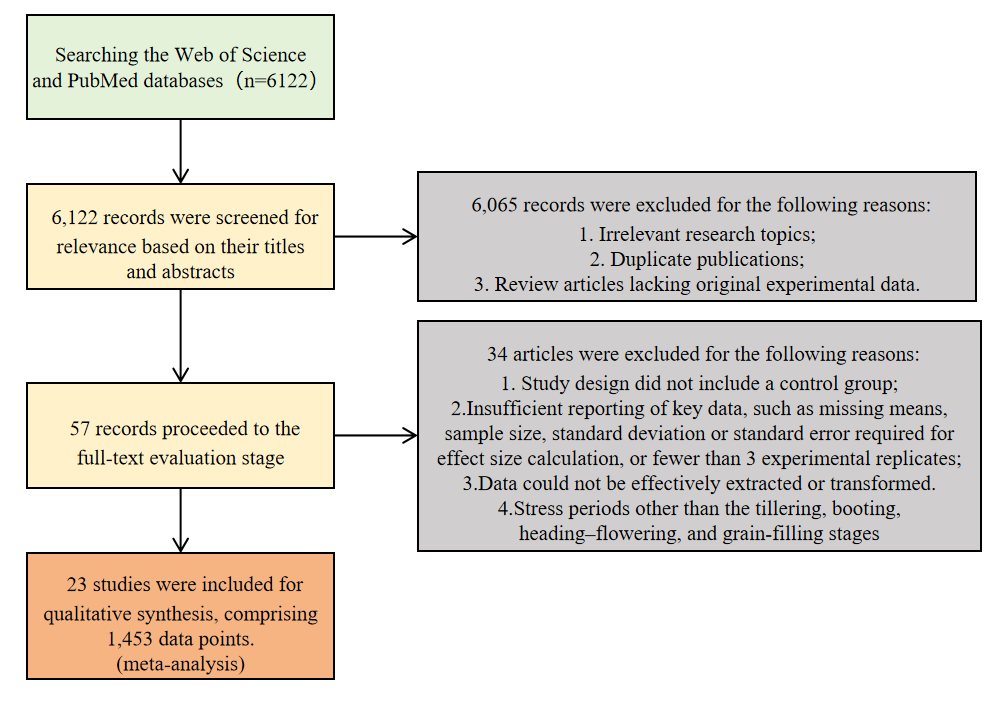


**Figure S1.** In the meta-analysis, a schematic diagram of the retrieval and screening process for 23 included literature is provided.


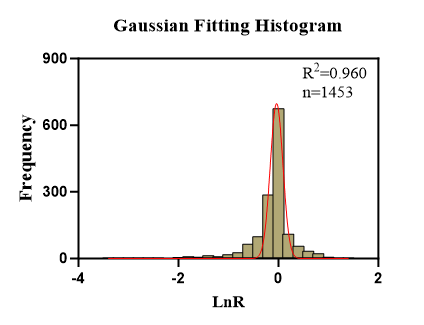


**Figure S2.** Gaussian-fitted histogram. Symmetry can be used to visually assess publication bias of the included studies.

**Figure S3.** Dose–response curves using 20 ℃ as the reference temperature.

**
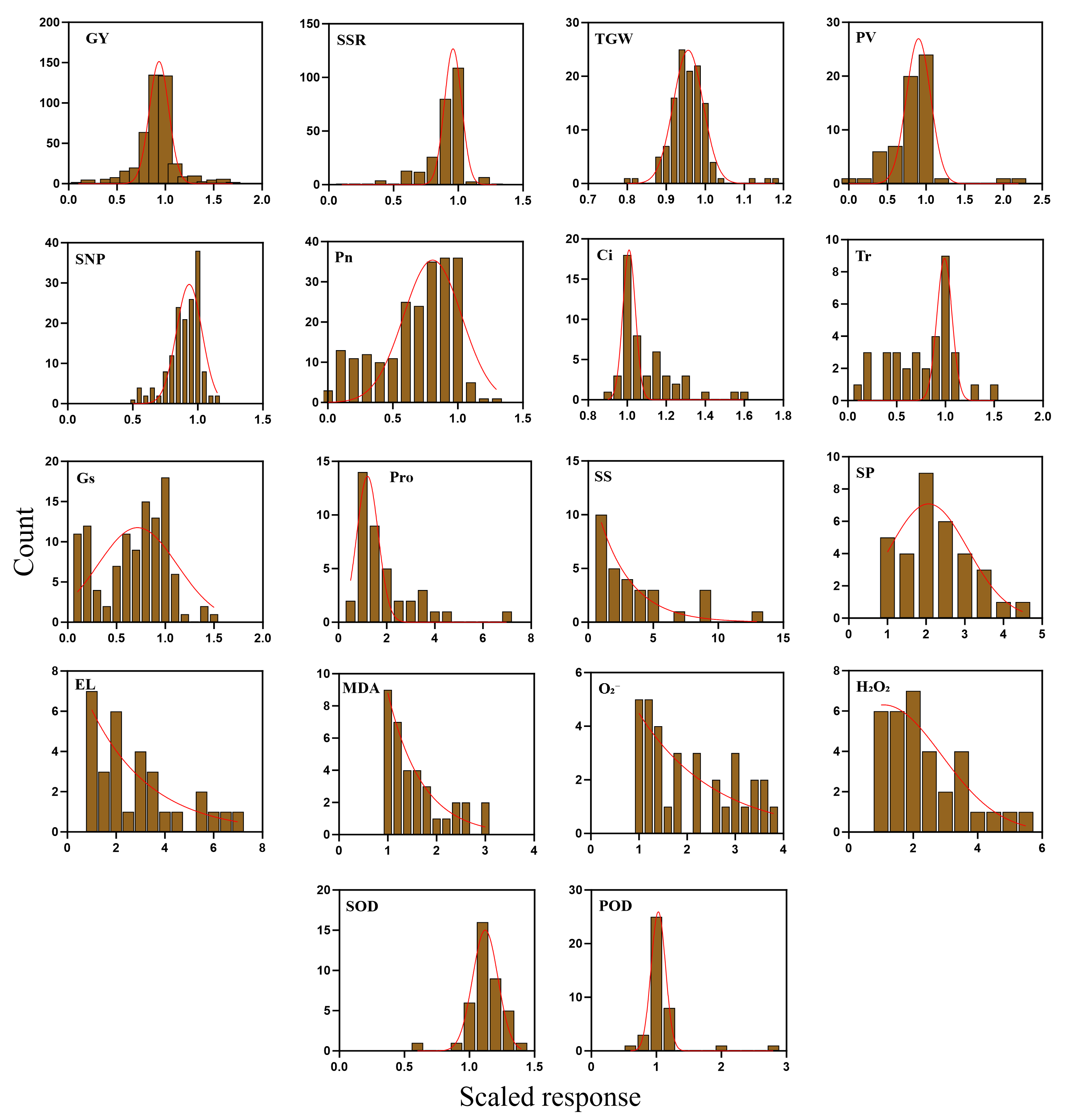
Figure S4.** Histogram of frequency distribution of standardized response variable.

**Table S1.** Assessment of between-study heterogeneity for effect sizes across indicators.

| Indicator | $I^{2}$ (%) | $\tau^{2}$ |
| --- | --- | --- |
| GY | 96.72 | 0.049 |
| SSR | 98.52 | 0.040 |
| TGW | 79.22 | 0.001 |
| PV | 96.42 | 0.077 |
| SNP | 96.84 | 0.008 |
| Pn | 99.26 | 0.187 |
| Ci | 95.63 | 0.009 |
| Tr | 98.48 | 0.296 |
| Gs | 99.50 | 0.558 |
| Pro | 94.13 | 0.082 |
| SS | 96.41 | 0.191 |
| SP | 84.04 | 0.035 |
| EL | 95.77 | 0.072 |
| MDA | 93.43 | 0.049 |
| O₂ | 71.16 | 0.012 |
| H₂O₂ | 97.01 | 0.067 |
| SOD | 91.89 | 0.008 |
| POD | 99.34 | 0.076 |

**Table S2.** Publish offset test results.

| Variable | Fail-safe-number | Prob(Chi-Square) |
| --- | --- | --- |
| GY | 34551.60 | 0.000 |
| SSR | 6953.50 | 0.000 |
| TGW | 409.90 | 0.335 |
| PV | 2147.20 | 0.000 |
| SNP | 5570.50 | 0.000 |
| Ci | 270.80 | 0.041 |
| Pn | 30170.20 | 0.000 |
| Gs | 12623.50 | 0.000 |
| Tr | 299.10 | 0.000 |
| Pro | 1024.20 | 0.005 |
| SS | 364.60 | 0.008 |
| SP | 744.60 | 0.507 |
| MDA | 308.40 | 0.901 |
| EL | 674.70 | 0.274 |
| O_2_- | 1598.00 | 0.224 |
| H2O2 | 950.40 | 0.376 |
| SOD | 1194.40 | 0.000 |
| POD | 310.00 | 0.000 |

**Table S3.** Subgroup analysis significance results.

| Variable | Stress Period | Stress Duration | Stress Temperature |
| --- | --- | --- | --- |
| GY | 0.001 | 0.001 | 0.001 |
| Pn | 0.005 | 0.001 | 0.001 |
